# Reducing multi-sensor data to a single time course that reveals experimental effects

**DOI:** 10.1186/1471-2202-14-122

**Published:** 2013-10-14

**Authors:** Aaron Schurger, Sebastien Marti, Stanislas Dehaene

**Affiliations:** 1 INSERM, Cognitive Neuroimaging Unit, Gif sur Yvette 91191, France; 2 Commissariat à l’Energie Atomique, Direction des Sciences du Vivant, I2BM, NeuroSpin center, Gif sur Yvette 91191, France; 3 Université Paris-Sud 11, Orsay 91405, France; 4 Collège de France, 11 Place Marcelin Berthelot, Paris 75005, France

**Keywords:** Spatial filter, Electroencephalography, Magnetoencephalography, Electrocorticography, Functional magnetic resonance imaging, Decoding, EEG, MEG, ECoG, fMRI

## Abstract

**Background:**

Multi-sensor technologies such as EEG, MEG, and ECoG result in high-dimensional data sets. Given the high temporal resolution of such techniques, scientific questions very often focus on the time-course of an experimental effect. In many studies, researchers focus on a single sensor or the average over a subset of sensors covering a “region of interest” (ROI). However, single-sensor or ROI analyses ignore the fact that the spatial focus of activity is constantly changing, and fail to make full use of the information distributed over the sensor array.

**Methods:**

We describe a technique that exploits the optimality and simplicity of matched spatial filters in order to reduce experimental effects in multivariate time series data to a single time course. Each (multi-sensor) time sample of each trial is replaced with its projection onto a spatial filter that is matched to an observed experimental effect, estimated from the remaining trials (Effect-Matched Spatial filtering, or EMS filtering). The resulting set of time courses (one per trial) can be used to reveal the temporal evolution of an experimental effect, which distinguishes this approach from techniques that reveal the temporal evolution of an anatomical source or region of interest.

**Results:**

We illustrate the technique with data from a dual-task experiment and use it to track the temporal evolution of brain activity during the psychological refractory period. We demonstrate its effectiveness in separating the means of two experimental conditions, and in significantly improving the signal-to-noise ratio at the single-trial level. It is fast to compute and results in readily-interpretable time courses and topographies. The technique can be applied to any data-analysis question that can be posed independently at each sensor, and we provide one example, using linear regression, that highlights the versatility of the technique.

**Conclusion:**

The approach described here combines established techniques in a way that strikes a balance between power, simplicity, speed of processing, and interpretability. We have used it to provide a direct view of parallel and serial processes in the human brain that previously could only be measured indirectly. An implementation of the technique in MatLab is freely available via the internet.

## Background

Many techniques for the measurement of neural activity result in multivariate time series. Methods such as electroencephalography (EEG), magnetoencephalography (MEG), electro-corticography (ECoG), and near-infrared spectroscopy (NIRS) may involve tens or even hundreds of sensors. Although all of these methods have some degree of spatial selectivity, there is also a significant amount of redundancy between different sensors: any given experimental effect, no matter how well localized, will normally appear in more than one sensor. With many sensors and potentially many possible experimental effects and interactions, one is confronted with the question of which sensors to subject to analysis and how to choose them. This is paramount in analyses where one is specifically interested in the within-trial time course of an experimental effect.

Perhaps the most widely-used approach is to select a single sensor or take the average over a contiguous cluster of sensors – a "region of interest" or ROI. Although the ROI approach is simple and readily interpretable, it does not take into account the distribution of activity across the sensor array. Nor does it account for situations where a given experimental effect appears in two or more non-contiguous regions, with potentially opposite signs. Thus many sensors that are sensitive to a given experimental effect may be left out of the ROI, and the sensors that are included in the ROI are all treated equally even though some may carry much more signal than others.

An ROI is a special case of a linear spatial weighting applied to the sensors [1], often referred to as a spatial filter, where the sensors belonging to the ROI have a weight of 1/*n* (*n* being the number of sensors belonging to the ROI) and all other sensors have a weight of zero. A number of techniques are available for deriving spatial filters so as to capture distinct signal sources in the data ([1] and see Discussion). Spatial filtering can be highly effective in detecting signal features in multi-sensor data, even at the single-trial level [2].

In signal processing a common technique for detecting the presence of a known signal, *s*, in noisy data, is to simply use *s* itself as a filter. Typically a different label, *h*, is used to refer to *s* when treated as a filter. In this context, *h* (= *s*) is referred to as a “matched filter” because the filter is matched to (identical to) the shape of the known signal that we are trying to detect, but that might be hidden in noise. Filtering is performed by simply correlating the filter, *h*, with a segment of noisy data, *x*. A matched filter is the optimal filter for revealing the presence of a known signal (i.e. maximizing the signal-to-noise ratio, or SNR), assuming independent and identically distributed (i.i.d) noise [3,4]. If the noisy data are in the form of an observed spatial topography (e.g. over an EEG montage) rather than a time series, then the filter is in effect a linear spatial weighting, and we apply the filter by taking the dot product (a.k.a. scalar product) of the filter and the noisy data (this operation is explained below). A *matched spatial filter* by itself is almost always sub-optimal in actual practice because the assumption of i.i.d noise is rarely, if ever, met. However, in many cases the use of a matched spatial filter alone will yield a significant *improvement* in SNR, and has practical advantages owing to its simplicity.

**Figure 1:**
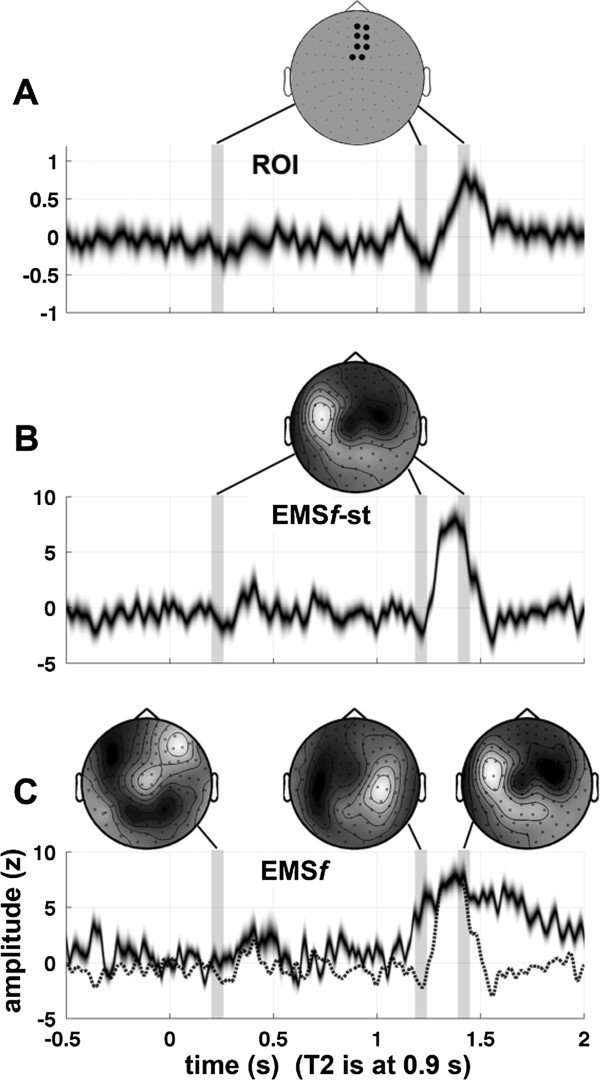
**Illustrative comparison of region of interest (ROI) and EMS filtering analyses applied to a representative subject.** The mean time course of an ROI **(A)**, fixed spatial filter **(B)**, and evolving spatial filter **(C)** for the difference between the *lag-9* and *control* conditions in the data from Marti et al (2012). Just above each of the panels is the topography of the average spatial filter within each of three different temporal windows: 0.25 to 0.30 sec, 1.20 to 1.25 sec, and 1.40 to 1.45 sec. Only the magnetometers are shown for clarity. Note that the ROI “spatial filter” is discrete and binary, and is identical at all time points in the epoch. The time course in **(B)** was derived using a stationary filter computed over the data in a specific time window (EMS*f*-st), with the spatial filter defined as the mean difference between the *lag-9* and *control* conditions between 1.35 and 1.45 sec. The grayscale error boundary extends to 99% confidence (on a *t* distribution, df = 9). Note that the difference is maximized in the time window over which the spatial filter was defined. Note also that, as in **(A)**, the spatial filter is identical at all time points, but that unlike **(A)**, the spatial filter is continuous-valued rather than discrete. The time course in **(C)** was derived from the output of canonical EMS filtering. The dashed line shows the time course of the stationary template used in panel **B (**for visual comparison with panel **B)**. Note in panel **C** that the spatial filters are continuous-valued and are also changing across time in the epoch (the spatial filter is computed independently at each time point in the epoch according to the objective function, which in this case is simply the difference between the *lag-9* and *control* conditions).

A given spatial filter can only capture the time course of activity from a single fixed vantage point. Experimental effects, on the other hand, almost never have the same distribution across the sensor array throughout the time course of a trial epoch. The sensor(s) that most strongly exhibit the effect will change over time, reflecting the spatio-temporal evolution of the underlying activity in the brain. In order to examine the time course of an experimental effect, the vantage point (i.e. the weighting applied to the sensors) has to change over time (see Figure 1C).

Here we propose a simple, powerful, and versatile technique that uses matched spatial filters^a^ in order to reduce epoched multi-sensor data to a single time course that tracks a given experimental effect across the timespan of each trial. Rather than being defined a-priori, the filters are estimated directly from the data itself, independently at each time point – hence the name “*effect-matched spatial filtering*” (*EMS* filtering). The “data” in this context are assumed to be a multi-channel time series with repeated trials (i.e. a three-dimensional matrix, *channel* x *sample* x *trial*). The goal is to reduce each trial’s data (a *channel* x *time* matrix) to a single time course that measures the magnitude of an experimental effect at each time point in the epoch, resulting in a *trial* x *time* matrix of *surrogate time courses*. These can be used to reveal the temporal evolution of an experimental effect, which distinguishes this approach from techniques such as principal components analysis (PCA) or independent components analysis (ICA) that reveal the temporal evolution of a fixed linear combination of the channels.

Because the method is driven by both the data and the data analysis question, and is applied separately at each time point, the resulting time series can be thought of as a functional reconstruction: instead of attempting to reconstruct the time course of an anatomical source, we reconstruct the time course of an experimental or behavioral effect (whose anatomical generators may change across time) in the original units (e.g. micro-volts or femto-tesla). We validate the method using MEG data from a previously-published study [5]. Part of this study involved measuring the latency of specific brain events in the average across groups of trials. Here we demonstrate how EMS filtering is able to estimate this latency on single trials and thus directly test for a trial-by-trial correspondence between the latency of brain events and reaction time.

### Use of EMS filtering to measure serial and parallel brain processes

We tested *EMS* filtering on data come from a prior study (Marti et al., 2012) that investigated the brain mechanisms of the psychological refractory period (PRP). The PRP is a behavioral phenomenon in which participants are slower to perform the second of two independent tasks when stimuli are presented in close succession [6]. In Marti et al.’s paradigm, participants were asked first to detect an auditory tone (high or low pitch, “task 1”) and then a visual letter (‘Y’ or ‘Z’, “task 2”). The experiment was designed to test the “router model” of the PRP [7] according to which two different stimuli, in this case the tone and the letter (T1 and T2 respectively), can be integrated in parallel at the sensory level, while the execution of task decisions requires access to a serial central stage of processing. When the execution of the two tasks overlaps in time, the second stimulus is integrated at the perceptual stage but has to wait in a sensory buffer while the first stimulus is processed at the serial stage. Once task 1 is completed, then T2 can access the serial stage and the second decision operation can be carried out. This model makes two important predictions regarding brain activity related to parallel and serial brain processing: (1) The onset of early sensory activity related to T2 should be time-locked to the presentation of T2 and prolonged at least until a decision is reached for task 1 – i.e. its duration, but not its latency, is expected to covary with the reaction time to T1 (RT1). (2) This should be followed by a second wave of activation, corresponding to the access to the serial stage. The latency of this second wave of activation, but not its duration, is expected to covary with RT1 (the converse of the prediction regarding the sensory stage).

We used EMS filtering to examine the precise relation between the duration of the sensory stage, latency of the central stage, and the duration of T1 processing, at the single-trial level, and to reconstruct T2-related main effects in the time-locked average. We demonstrate the efficacy of EMS filtering in improving the signal to noise ratio of individual trials and in revealing the time course of an experimental effect (the difference between two conditions) in the time-locked average. We also use EMS filtering to test specific predictions derived from the router model [7] regarding parallel and serial processing in the brain.

## Methods

### Applying a spatial filter: signal projection

Throughout the manuscript we use the word “projection” in the sense of “orthogonal projection onto a line”, which is equivalent to taking the dot product of two vectors, *χ* and *w*. If *χ* is an observed topography and *w* is a spatial filter, then we apply the filter by taking the dot product of *χ* and *w*, i.e. by projecting *χ* onto *w*. The dot product (or scalar product) involves multiplying the two vectors element-by-element, and then taking the sum of the result, expressed as a single scalar value. It is closely related to correlation: for a given *w*, the more closely *χ* resembles *w* the larger the dot product (keeping the norm of *χ* constant). Setting the filter, *w*, to unit length (‖*w*‖ *= 1*), has the advantage of preserving the measurement units of any data that are projected onto it: If *χ* is a vector of measurements in micro-volts, then the projection of *χ* onto *w* will result in a single scalar value also expressed in micro-volts. In summary: A spatial filter is simply a set of coefficients (one for each sensor) in the form of a vector, which can be visualized as a topographical pattern over the sensor array. The filter is applied by taking the dot product of the data (an observed topography) and the filter, resulting in a single scalar value that pools activity from all of the sensors.

### Effect-matched spatial filtering

When analyzing multivariate signal data, the goal is most often to address a particular scientific question, for example “what is the time course of the experimental effect (condition A) with respect to that of the control condition (condition B) in my data?” This question could apply to EEG, MEG, ECoG, or other multi-sensor data. One can estimate the effect across time in the trial epoch by taking the mean (across trials) of the data belonging to condition A, the mean (across trials) of the data belonging to condition B, and then computing the difference between them. This has to be done separately for each sensor, resulting in as many time courses as there are sensors, with the experimental result (if there is one) distributed among them. We want to summarize each trial’s data (a *channel* x *time* matrix) in a single time course, using a weighted combination of the sensors (spatial filter) that is tuned to detect the experimental effect.

The canonical matched-filtering approach would be to start with a *known* topographical pattern corresponding to “the difference between condition *A* and condition *B*”, and use that topographical pattern as a spatial filter. However, in an experimental setting, we do not know what “the difference between condition *A* and condition *B*” is supposed to be at each time point in the trial epoch (or at any time point, for that matter). The solution given by our approach is to measure the experimental effect in the data itself, and use the resulting topographical pattern as a spatial filter. The filter is set to unit length (by dividing by its own norm) in order to preserve the units of the data that will be projected onto it.

We also want the resulting time course to “follow” the experimental effect across time. Hence the procedure is repeated separately and indpendently for each sample in the trial resulting in a *filter set* (one spatial filter for each time point) as well as a single “surrogate time-course” per trial (the result of applying the filter set to each trial’s data).

**Figure 2:**
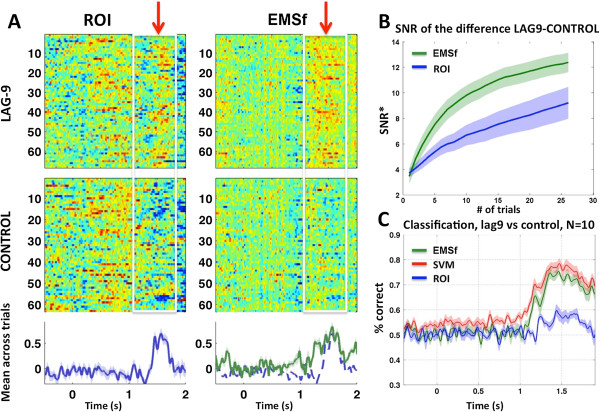
**EMS filtering improves the quality of single-trial time courses.** Panel **(A)** presents data from a representative subject, comparing the ROI method (left) to the EMS filtering method (right). There are two columns of two raster plots, with time on the horizontal and trial # on the vertical. Amplitude is coded in color, going from blue (negative) to green (zero) to red (positive), and the color axis is scaled to the minimum and maximum amplitude in each data matrix. Below each pair of raster plots (top: *lag-9* condition; bottom: *control* condition) is the time course of the difference between the means of the two conditions, mean(*lag-9*) – mean(*control*). No smoothing was applied to the images. Panel **(B)** shows the estimated mean signal-to-noise ratio as a function of the number of trials averaged together, for the ROI (blue) and EMS filtering (green) methods. Panel **(C)** shows the mean performance (10 subjects) of a univariate Gaussian naïve-Bayes (GNB) classifier tested on the output of a nested EMS filtering procedure (green; see methods). The performance of a GNB classifier applied to the mean over the ROI (blue) and the performance of a linear support-vector machine (red) are shown for comparison. Notice that the performance of a univariate decision rule (GNB) applied to the output of EMS filtering is comparable to the performance of a multivariate linear SVM applied to the original sensor data.

In order to avoid circularity in the procedure [8,9], the data that are projected onto the filter set must be independent of the data used to derive the filter set. The best estimate of the filter set, while avoiding circularity, is derived based on all of the data except for one trial that we set aside and project onto the filter. [We must leave out at least one trial in order to avoid circularity, and the more trials we leave out the fewer trials remain on which to base our estimate of the filter.] Thus we use a simple leave-one-out (LOO) or leave-one-out-per-class (LOOPC) procedure: we iteratively project each trial’s data onto the spatial filter-set derived from all of the other trials. The LOO procedure renders the surrogate time courses unbiased (see [9] and Additional file 1), and hence more readily interpretable – wherever there really is no effect to be found, the experimental effect in the surrogate time courses is expected to be zero (^b^ and Additional file 1). In addition to being a method of cross validation, the LOO procedure, when used in this way, has the effect of attenuating trial-specific noise, resulting in a higher SNR in the average across trials (Figure 2B).

### EMS filtering algorithm

(For a formal mathematical description of EMS filtering, see Appendix A).

Consider the data-analysis question posed above: “what is the time course of the experimental effect (condition A) with respect to that of the control condition (condition B) in my data?” We want to summarize this kind of experimental result in a single time course, using a weighted combination of the sensors (spatial filter) that yields a higher signal-to-noise ratio than any single sensor. We also want the resulting time course to “follow” the experimental effect across time.

The procedure is simple (the two steps below are applied separately and independently at each time point). Let ⟨χ⟩ denote the mean over the elements of the vector χ. Let χ_A_ be a vector of measurements at a single channel and single time point, for all trials belonging to condition *A*, and likewise for χ_B_. Let χ^(k)^ be a vector of measurements at a single channel and single time point, for all trials *except* for trial *k* (i.e. with the *k*^th^ trial left out if *x* contains the *k*^th^ trial). Then

1. For each trial, *k*:

a. Compute 〈*x*_*A*_^(*k*)^〉 - 〈*x*_*B*_^(*k*)^〉 at each channel and treat the resulting vector, *w* (one scalar value per channel), as a spatial filter.

b. Set *w* to unit length, i.e. let $\hat{w}=\frac{w}{∥w∥}$.

c. Use $\hat{w}$ as a spatial filter for the left-out trial (trial *k*) by taking the dot product of w^' (a 1 x *channel* vector) and the data (a *channel* x 1 vector). This results in a single *surrogate value*, sk=w^'xk.

2. Compute ⟨S_*A*_⟩•⟨S_*B*_⟩ over the resulting surrogate values.

The results (one for each time point in the trial epoch), when strung together in temporal order, yield a single time course that gives an answer to the data analysis question, in this case “what is the time course of the experimental effect (condition A) with respect to that of the control condition (condition B) in my data?” The entire set of surrogate values belonging to a single trial is referred to as a *surrogate time course*, and the entire set of surrogate time courses (one per trial) is a *trial* x *time* matrix denoted by the symbol *S* (lowercase *s* is used to denote a single surrogate value).

Although the procedure above is defined in an iterative, leave-one-out (LOO) fashion, note that a leave-one-out-per-condition (LOOPC) procedure can be used if there are an equal number of trials in each condition. Group-level analyses can be applied to the results from each subject using standard methods. The algorithm can also be applied across subjects, where ⟨χ_*A*_⟩ and ⟨*χ*_B_⟩ are pre-computed separately for each subject, in which case a leave-one-*subject*-out procedure should be used.

In order to generalize to data-analysis questions other than the example given above (the difference between the means of two experimental conditions), we refer to the computational operation corresponding to the data analysis question (e.g. ⟨χ_*A*_⟩ - ⟨χ_B_⟩) as the *objective function*. Hence, in the general version of the algorithm, ⟨χ_*A*_⟩ - ⟨χ_B_⟩ in step 1 above is replaced by *F*(*X*,*y*), where *F* is the objective function, *X* is the data matrix (*channel* x *sample* x *trial*) and *y* is a vector of condition labels (one for each trial). The objective function is simply a computational representation of the data analysis question, which might, for example, concern the relationship between the sensor data and a behavioral variable such as reaction-time. The MatLab toolbox that we provide includes standard objective functions, but also allows for the objective function to be user-defined. This makes the method highly versatile (see Discussion).

For some purposes one might want to compute a single spatial filter and apply the same filter at all time points in the trial epoch, and this is also supported by our toolbox. By doing so it is possible to examine the time course of a specific stationary topography. For instance, if one was interested in the time course of the P300 component of the ERP in EEG data, one could compute a single topography over a time window centered on the peak of the P300. Applying the topography as a spatial filter would result in a single time course highlighting the onset, peak, and cutoff of that specific spatial pattern. Another scenario where one might use EMS filtering in this way would be to estimate the “readiness potential” (RP) [10] – a slow buildup of neuronal activity that precedes self-initiated movements – from multi-sensor data. For this purpose one could use an objective function that computes the difference between the amplitude at ~100 ms prior to movement onset with that at ~500 ms prior to movement. Sensors at which the mean amplitude is changing over that time interval will be weighted more heavily than sensors at which the mean amplitude does not vary over that interval. The resulting filter should expose the time course of the (functionally-defined) RP more accurately than any single sensor.

### Non-independence of the surrogate time courses

**Figure 3:**
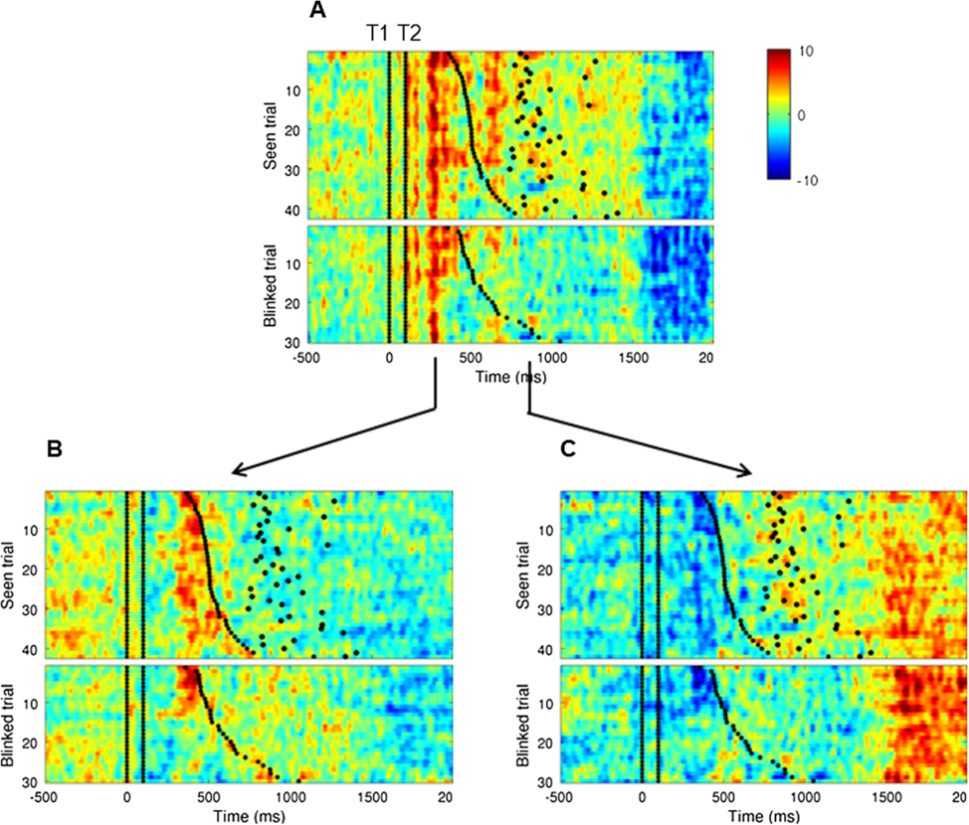
**Applying EMS filtering to test predictions regarding the psychological refractory period.** Data presented are for Seen and Unseen trials from the Lag 1 condition sorted according to RT1 and time locked to T2. The spatial filter used in the analysis was computed by subtracting Lag 1 (seen) and Control conditions. Each panel represents trials as a function of time sorted according to the speed of RT1, after using an evolving spatial filter **(A)**, or stationary spatial filters computed at specific latencies: the averaged amplitude between 200–300 ms **(B)** or 700–900 ms **(C)** after T2 onset. For subsequent analysis, we refer to these two components as the *early* and *late* components, respectively. Previously (Marti et al., 2012) these had been labeled according to their latencies in the lag 9 condition, i.e. M250 and M550. Black dots represent RT1 (small) and RT2 (big). Black lines represent T1 onset (0 ms) and T2 onset (100 ms).

**Figure 4:**
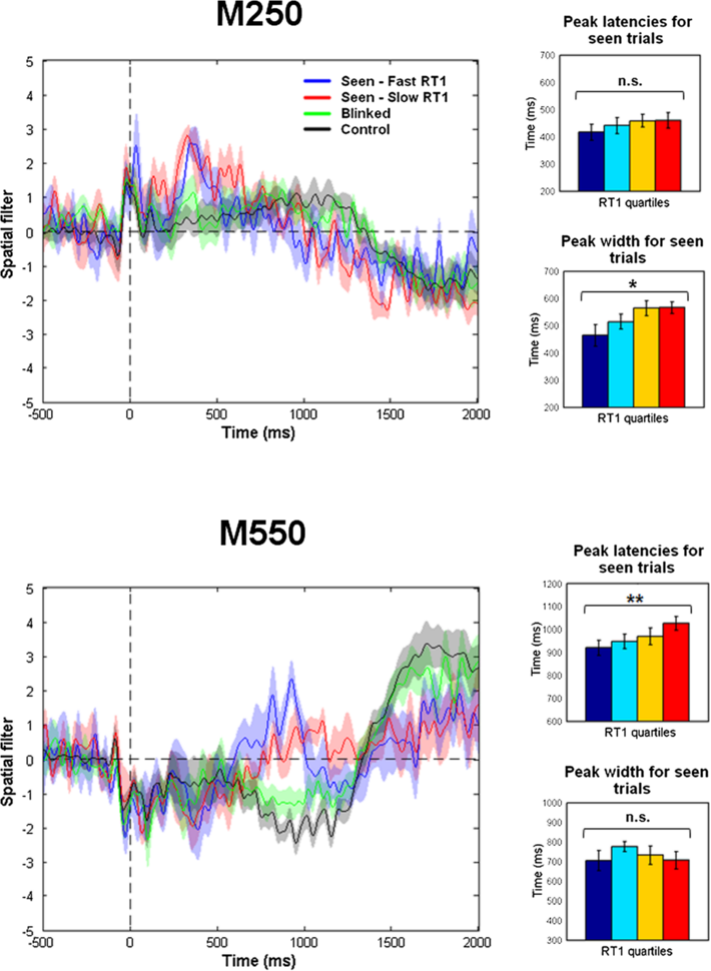
**Average across subjects for the *early *(M250) and *late *(M550) components for lag 1 and control conditions, time locked to T2.** Seen trials were split according to the speed of RT1. Trials where RT1 was smaller than the first quartile was classified as fast (blue line) and trials above the third quartile were classified as slow (red line). Blinked and Control trials are represented in green and black respectively. Over the group, the M250 was time locked to the onset of the stimulus and larger for slow versus fast RT1, while the M550 was delayed for slow versus fast RT1 trials. Bar plots on the right represent the group averaged peak latency and duration of the M250 (upper part) and the M550 (lower part) for seen trials in lag 1 condition. Each bar represent a quartile of RT1 from fast (blue) to slow (red) reaction times. An ANOVA with RT1 quartiles as a within-subject factor revealed a significant effect on the duration of the M250 but not on its latency. It was the opposite for the M550: a significant effect was observed on the latency but not on the duration.

Although each trial is independent of the spatial filter onto which it is projected, the spatial filters themselves (one per trial) are not independent of one another since they are all based on nearly the same set of trials. Therefore, the resulting surrogate time courses are not independent of one another. However, if they are grouped by condition and averaged together for each subject, then the resulting averages for each subject *are* independent of one another, and so classical closed-form statistics can be used. This is the most likely use of EMS filtering, and so the non-independence of the surrogate time courses will most often not be a concern. On the other hand, if one wants to perform statistics on the aggregate over surrogate time courses *within* a single subject, or if EMS filtering is applied at the group level (i.e. by replacing “trial” with “subject” in the data matrix), then resampling tests, such as the permutation test or bootstrap, should be used. The non-independence of the surrogate time courses is not a concern in the case of within-subject single-trial analyses (as in Figure 3) that do not involve averaging across subsets of the surrogate time courses, or that are concerned with the latency or duration of an effect (as in Figure 4) rather than the amplitude.

### Multiple comparisons

EMS filtering eliminates the need for multiple comparisons corrections across the sensor array. However, it does not eliminate the need to correct for multiple comparisons across time (samples) in the trial epoch. The same practices normally applied in the context of time-locked averaging of a single sensor or ROI are appropriate.

### Data set: MEG experiment (Marti et al. 2012)

All details about participants and MEG recordings are reported in Marti et al. (2012). Briefly, ten subjects were included in MEG analyses. All subjects were naïve to the task, had normal or corrected-to-normal vision, and gave written informed consent to participate.

### Stimuli and apparatus

All participants performed a dual-task paradigm in which the first target was a monotonic sound presented to both ears and the second was a letter of the alphabet presented visually. The first target (auditory) could be a high pitch (1100 Hz) or a low pitch (1000 Hz) and was presented for 84 ms. The second target (visual) was either the letter "Y" or the letter "Z" presented in black on a white background, (0.64 º of visual angle). The target letter was embedded in a visual stream of 12 random black letters used as distractors. Each letter was presented at the center of the screen for 34 ms with an inter-stimulus interval of 66 ms. The target sound (T1) was always synchronized to the third distractor and followed by the second target (T2) after a variable inter-target lag of 100, 200, 400 or 900 ms. In a fifth condition, T2 was replaced by a non-target letter of the alphabet (“distracter” or “control” condition). Participants were instructed (1) to respond as fast as possible first to the sound and then to the letter, (2) to respond as soon as the corresponding stimulus appeared, thus avoiding "grouped responses", and (3) that the second stimulus would occasionally be absent, in which case they should simply not perform the second task.

The experiment consisted of two training blocks of 20 trials each, one to practice the auditory task and the other one to practice the visual task, followed by 5 experimental blocks. In four of these experimental blocks, participants performed 100 trials of the dual-task and in one block they performed 50 trials of only the visual task while they had to listen passively to the sound (T1-irrelevant condition). Thus, a maximum of 80 trials per inter-target lag were recorded.

### MEG recordings and pre-processing

While subjects performed the cognitive tasks, we continuously recorded brain activity using a 306-channel whole-head magnetoencephalography system (Elekta Neuromag®) with 102 gradiometers and 102 pairs of orthogonally oriented planar gradiometers. The subject's head position was measured at the beginning of each run and this information was used during data pre-processing to compensate for differences in head position between runs. Electro-oculogram (EOG) and electrocardiogram (ECG) were recorded simultaneously for offline rejection of eye movements and cardiac artifacts. Signal Space Separation, head movement compensation, and interpolation of bad channels were applied using the MaxFilter Software (Elekta Neuromag®). Epoching, trial rejection, and baseline correction were then applied using the Fieldtrip software package (http://fieldtrip.fcdonders.nl/). Independent components analysis (ICA) was used to identify and remove ocular and cardiac artifacts. Data for each of the three subsets of sensors (one array of magnetometers and two arrays of gradiometers) were separately converted to Z-scores by normalizing to the mean and standard deviation over the entire data set for that sensor type. This is necessary because data from magnetometers and gradiometers are expressed in different units and the range of values differs by an order of magnitude.

### Measure of signal-to-noise ratio

To estimate the signal-to-noise ratio (SNR; Figure 2A and B) we first identified the time window of the maximum difference between the *lag-9* and *control* conditions, which was in the time range 1.4 to 1.7 sec (the onset of the target stimulus, T2, was at 0.9 sec). We chose the interval from -0.5 to -0.3 sec as the baseline (noise) interval. SNR was then computed as the root-mean-squared difference between the *lag-9* and *control* conditions, in the selected time window, divided by the standard deviation of the difference in the baseline time interval. This value was then log transformed to express it in decibels. More precisely:

$$\mathrm{SNR}=10\log_{10}{\left(\mathrm{RM}S_{s}\mathrm{STD}\right)}^{2}$$

Where

$$\mathrm{RM}S_{s}=\sqrt{\frac{\sum_{i\in S}d_{i}^{2}}{n_{s}}},\mathrm{ST}D_{N}=\;\sqrt{\frac{\sum_{i\in N}{\left(d_{i},-,\bar{d}\right)}^{2}}{n_{N}-1}}$$,

*S* refers to the set of samples belonging to the “signal” interval (1.4 to 1.7 sec), *N* refers to the set of samples belonging to the “noise” (or baseline) interval (-0.5 to -0.3 sec), *n*_*S*_ is the number of samples in the “signal” interval, *n*_*N*_ is the number of samples in the “noise” interval, and *d* is the difference between the mean over trials belonging to the *lag-9* condition and the mean over trials belonging to the *control* condition.

To estimate the SNR for different numbers of trials, for each *n* in 1,…,25 we computed the SNR for a random selection of *n* trials. We repeated this 100 times for each value of *n* in order to arrive at a more precise estimate (Figure 2B).

### Decoding analysis

In Figure 2C we present the results of an analysis where we attempted to classify each time point in each trial as belonging to either the *lag-9* or the *control* condition. For this analysis we used a nested EMS filtering procedure and a Gaussian naïve-Bayes (GNB) classifier. For each iteration of the outer loop, we set aside two trials, one from each condition. Then, for the inner loop, we performed EMS filtering on the remaining trials in order to estimate the mean, among the resulting surrogate measures, for each condition. If there were more trials in one condition than in the other, then we randomly selected a subset of the trials from the condition with the greater number of trials, so as to have an equal number of trials per condition.

For each time point in each of the two outer-loop left-out trials, we computed its posterior probability given the mean and variance of the surrogate measures, output from the inner loop, for each of the two categories (*lag-9*, *control*). The category with the highest probability was taken as the classifier’s decision. The resulting time course of classification accuracy was then smoothed with a 50 ms sliding window before averaging across subjects. The same procedure was used for decoding based on the ROI, except that at each iteration of the EMS filtering algorithm, the spatial filter was replaced by the ROI (Figure 2C): Each sensor belonging to the ROI was assigned the weight 1, all other sensors were assigned a weight of zero, and then the vector was set to unit length by dividing by its norm.

For comparison, we also performed decoding using a linear support vector machine (SVM) with five-fold cross validation (L2 loss function, L2 penalty, penalty parameter (C) = 1). As with the other decoding analyses, we equalized the number of trials in each condition by selecting a random subset of trials from the condition with a greater number of trials. We ran the SVM five times for each subject and averaged these together and pooled the variance (for Figure 2C). For this analysis we used the SciKit Learn toolbox for Python [11] (available at http://scikit-learn.org).

## Results and discussion

### Results

#### Demonstrating the efficacy of EMS filtering

##### Advantages of EMS filtering at the single-trial level

Figure 2A presents the results obtained for a single subject and allows a direct comparison between the ROI approach and EMS filtering. EMS filtering appears to reveal the presence of the second target at the single trial level more clearly than does the ROI. In testing this more formally, we found that as the number of trials increases, EMS filtering significantly improves the signal-to-noise ratio (SNR) as compared to the ROI method (p < 0.05 for each trial count between 3 and 25; p < 0.01 for each trial count between 4 and 19; one-sided Wilcoxon signed rank test; Figure 2B).

The above conclusion is of course dependent on the choice of ROI, and in particular on how well the ROI targeted the effect of interest. The ROI that we used (see Figure 1A) was chosen using a cluster-based permutation test [12] applied to the *lag-9* versus *control* conditions. This test revealed a large cluster of latitudinal gradiometers with a significant difference between the two conditions at around 550 ms after T2, and the 8 sensors in the center of this cluster were chosen as the ROI. There was no other significant cluster in this time window. Technically, because the ROI was chosen using the same data on which the SNR and decoding results were computed, this introduces some circularity in the ROI-based analyses. We intentionally allowed this since it can only confer an advantage on the ROI method, and thus counts as more conservative test of EMS filtering.

To further test the quality of single trial data we attempted to classify each time point in each trial as belonging to either the *lag-9* or the *control* condition, based only on the one-dimensional output of the EMS filtering algorithm. We nested the EMS filtering procedure inside of the classifier cross-validation so that the training and test data sets were always disjoint (see Methods). A univariate GNB classifier applied to the output of EMS filtering (EMSf-GNB) reached a peak average performance of greater than 70% correct over the time range of 1.35 sec to 1.45 sec (ranging from 68% to 86%; Figure 2C, green line). For comparison we applied the same classifier to the average over an eight-channel region of interest (ROI-GNB) defined based on separate data (Figure 2C, blue line), and also applied a linear support-vector machine (SVM) to the original multi-sensor data (Figure 2C, red line). Although the SVM performed slightly better than EMSf-GNB, remarkably, discrimination of the two categories based on the univariate output of EMS filtering was comparable to the performance of the SVM, and both were markedly better than ROI-GNB (Figure 2C). Note that while the SVM yields slightly better separability of the two classes at the single-trial level, this may come at the expense of the “interpretability” of the topography of the weight vectors (see Discussion).

### Advantages of EMS filtering at the group level

**Figure 5:**
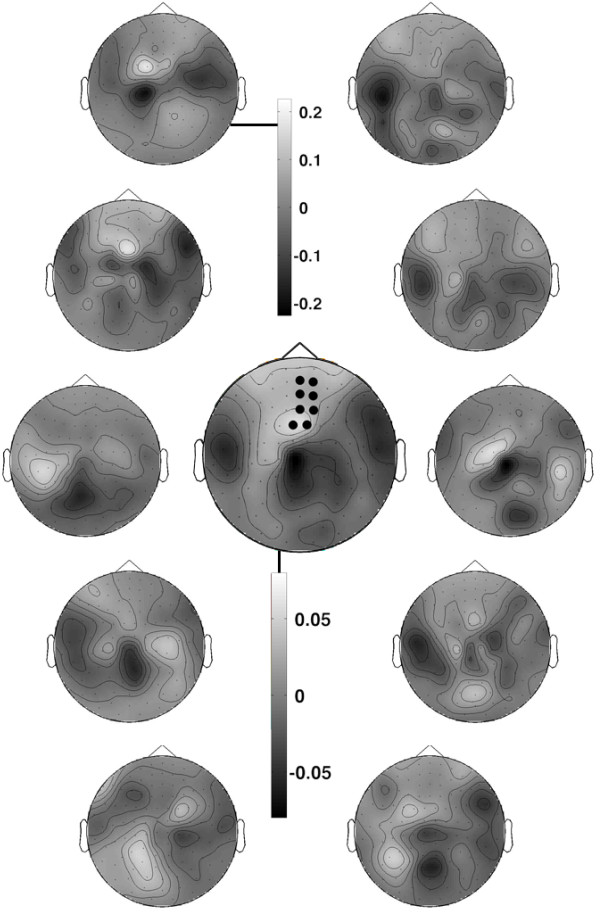
**Spatial filters for the same experimental effect are different for individual subjects.** The topography of the spatial filters for each of the 10 subjects used to produce Figure 1B (a stationary template was used for each subject). Only the latitudinal gradiometers are shown for clarity. The average topography over the 10 subjects is shown in the center, exposing the cluster that was identified using FieldTrip’s cluster-based permutation test [12], and used to define the region of interest (ROI; black dots). Each of the 10 subject-specific topographies (including the magnetometers and latitudinal gradiometers, not shown) yields a time course that maximally separates the means of the *lag-9* and *control* conditions *for that subject*. It is evident that, while the ROI captures a region that tends to have a higher amplitude signal on average across subjects, it is not an especially prominent region of activity for any individual subject. EMS filtering thus factors out anatomical variability across subjects in order to focus specifically on the time course of the experimental effect.

The algorithm is not only able to increase the quality of single-trial data but it also has two important advantages at the group level compared to standard subject averaging. First, because the spatial filter evolves across time (Figure 1C), the resulting surrogate time courses are specific to an experimental manipulation. By comparison, the ROI approach gives the time course of a fixed subset of sensors and is thus blind to the evolution of the topography in sensor space over the time span of the trial. Second, the matrix of spatial filters is unique for each subject (Figure 5), thereby factoring out anatomical variability across subjects and focusing specifically on the time course of the experimental effect.

### Revealing brain mechanisms of dual-task interference using EMS filtering

Figure 3A shows the result of EMS filtering applied to data from the *lag-1* condition for a single subject. We used a spatial filter computed from the subtraction *Seen* (*lag-1*) minus *Control* (i.e. target absent) and applied this filter to *Seen* and *Blinked* trials in the *lag-1* condition. In the average over the group, as well as for this particular subject, we identified two events in the resulting time course, one peaking at around 200–300 ms and another between 700 and 900 ms after T2 onset. We will refer to these as the *early* and *late* events, respectively. [These are presumed to correspond to the M250 and M550 components identified in Marti et al. (2012). However, we avoid naming these components in terms of their latency because the latency of the late component is highly variable due to the PRP effect – subject to the T2-T1 lag.] Note that the difference in the latency of the late event between the *lag-9* and the *lag-1* conditions was expected and simply corresponds to the PRP effect.

As can be seen in Figure 3B, the early event was time locked to T2 onset and its duration closely followed RT1. This was confirmed at the group level (Figure 4) by an ANOVA with quartiles of RT1 as a within-subject factor: the duration of the early component was strongly influenced by RT1 (F(3,27) [SD3] = 4.12; p < 0.05). This matches the prediction of the model regarding the existence and the properties of a perceptual buffer: T2 enters the buffer at a fixed time relative to T2 onset, but is held at this stage for a duration proportional to the central stage processing of T1 (and hence to RT1) before gaining access to the central stage. By contrast, the results for the late event (Figure 4 bottom) matched the properties of a serial central stage: the peak of the event, but not its duration, was influenced by RT1 (F(3,27) = 5.28; p < 0.01, Figure 4). This is consistent with the model according to which T2 enters the central stage only after task 1 completion, with a latency proportional to RT1.

Thus, EMS filtering afforded a unique view of the interference between the two tasks: we were able to investigate the precise relation between T1 processing and T2 processing both at the single trial level and at the group level. These results extend those obtained by S Marti, M Sigman and S Dehaene [6] by revealing both between- and within-subjects experimental effects. We found that the first MEG event that distinguished target from distractor stimuli matched our predictions regarding the existence of a sensory buffer during the PRP. It also shows a direct relation on a trial by trial basis between RT1 and the onset of the late event related to the conscious perception of T2.

### Performance

Since different software was used for the EMS filtering and SVM analyses (MatLab and Python, respectively), performance comparisons can only be considered descriptive and approximate. All analyses were tested on a Dell Precision T3400 PC (64-bit dual Intel Core-2 Duo CPU E8500 3.16 GHz, 6144 KB cache) running Ubuntu Linux 10.04. All processes were single-threaded, so only one processor was used. In all cases, results are reported for the *lag-9* versus *control* conditions (classification or contrast between means) with ~ 30 to 75 trials per condition per subject. The mean processing time of the SVM analysis with five-fold cross validation was ~ 1 to 2 minutes per subject (avg 1.44 seconds per trial, +/- 0.37s stdev). The mean running time of the SVM analysis with leave-one-out cross validation was ~ 6 to 8 minutes per subject (avg 6.86 seconds per trial, +/- 2.86s stdev). The mean running time of leave-one-out EMS filtering was ~ 2 to 3 seconds per subject (avg 0.042 seconds per trial, +/- 0.002s stdev), and varied linearly with the number of trials.

## Discussion

A number of techniques are available for deriving graded linear combinations of sensors so as to capture distinct sources of variance. Data-driven techniques such as principal component analysis (PCA) and independent component analysis (ICA) [13,14] decompose the data into separate “components” or "virtual sensors" whose time course is computed as a weighted sum of the real sensor output. These can be powerful techniques, but there is no way to know a priori which component(s), if any, will carry a particular experimental effect – it is up to the user to identify the component(s) of interest, and in most cases, the components that carry the most variance are artifacts. This can be useful for isolating and eliminating signal artifacts, such as eye blinks in EEG data [15]. However, when the objective is to isolate the most *relevant* sources of variance, rather than the most *pronounced* sources of variance, these methods are not ideal.

Hypothesis-driven methods, such as the covariance analysis [16,17] (not to be confused with the analysis of covariance, or ANCOVA), partial least squares (PLS) regression [18], and Fisher’s linear discriminant (FLD) try to isolate sources of variance that correspond to differences between experimental conditions. Other relations, however, such as interaction effects or correlation with an experimental or behavioral parameter cannot readily be addressed. Also, in the case of PLS, if more than two conditions are involved, e.g. *A*, *B*, and *C*, then it is difficult to know a priori which differences will be associated with each “latent variable” (the term used for "component" or "source" in PLS parlance). For example one latent variable might correspond to *A* versus *B & C*, and another to *B* versus *C*, while none corresponds to *A* < *B* < *C*, which, for the sake of argument, happens to be the effect of interest.

The metric known as “global field power” (GFP) [19], although technically not a spatial-filtering method, can be used to summarize multi-sensor data in a single time course, and can be applied to a derived measure such as the difference between two means. This provides a useful summary measure, but it is strictly an aggregate measure, and is not defined at the single-trial level. More recently, multiple regression has been used to track the time course of specific components of the stimulus-locked response [6,20,21]. However, because the regressors are derived empirically (from a separate data set), multi-collinearity can sometimes preclude its use (see Discussion). Two related methods, denoising source separation (DSS) [22,23] and common spatial patterns (CSP) [24,25], are also widely used, but primarily in brain-computer interface (BCI) applications. These methods are more complicated than the simple matched-filtering approach advocated here, and, like all of the methods discussed above (apart from GFP), are oriented towards extracting signal components (or sources) rather than experimental effects.

In the present study we propose a method capable of revealing the time course of experimental effects at the single-trial level and illustrate its potential by investigating serial and parallel processing in the brain. The algorithm presents several advantages compared to standard methods applied in human electrophysiology: EMS filtering (1) reduces high-dimensional data to one dimension, functionally determined by experimental conditions, (2) increases the SNR for single-trial data, (3) avoids the problem of anatomical variability when averaging across subjects, (4) is optimal in maximizing the difference between the means of two experimental conditions (see Appendix B), and (5) yields weight vectors (spatial filters) that are directly interpretable as topographies over the sensor array.

EMS filtering attenuates trial-specific noise by projecting the data from each trial onto a matrix of spatial filters derived from all of the other trials. In this sense EMS filtering is analogous to the method of “sensor noise suppression” (SNS) [26], except that it operates across trials rather than across sensors. Thus it might also be appropriate to refer to EMS filtering as a method of “trial noise suppression” (c.f. A de Cheveigné and JZ Simon [22]). An additional output of the method is a matrix of spatial filters, one for each time point in the trial epoch, whose topography and temporal evolution are also informative. As a complement to the analyses reported here, we also applied methods for identifying and visualizing stable states and transitions in the temporal evolution of the spatial filters (Additional file 2 and c.f. D Lehmann, H Ozaki and I Pal [27]).

Applying EMS filtering to the data of Marti et al. (2012) revealed an event at around 200–300 ms after T2, that matched the properties of a parallel perceptual buffer as described by the router model of the PRP [7]. It was selective and time locked to the second target but its duration was correlated with RT1. We also identified a later component peaking around 700–900 ms that had properties of a serial central stage: its onset was delayed by the execution of the first task while its duration remained unaffected. Hence, the use of EMS filtering allowed us to reveal new experimental findings that previously could only be inferred indirectly.

### Comparison with other methods

#### EMS filtering versus the ROI approach

Throughout the present paper, we have directly compared the ROI approach to EMS filtering. It is important to note that the use of one or the other depends on the scientific question being addressed. If the question concerns a specific region of the brain, then an ROI or projection onto a fixed anatomical source might be appropriate. However, if the question is about a specific experimental effect, then EMS filtering is a more appropriate tool because it tries to maximally reveal that effect separately at each time point by pooling over all sensors. We have shown that the method significantly increases the signal to noise ratio, especially when the number of trials is relatively small. We have also shown that a very simple univariate classifier (GNB) can perform well at discriminating between the *lag-9* and *control* conditions, based only on the one-dimensional output of EMS filtering, even without taking the noise covariance into account.

#### EMS filtering versus multiple regression with spatial templates

[5,20,28] used multiple regression in order to track the time course of different “components” of the stimulus-locked response on the very same data set. The topographies at specific peaks in the global field power for the *lag-9* condition were used as regressors in a multiple regression applied to the data from the other conditions. Multiple regression becomes problematic when the same variance can be accounted for by more than one regressor. However, if there is no multicollinearity in the predictors, multiple regression can potentially provide a better prediction of the relationship between the variables compared to either simple regression or spatial filtering. In addition, in Marti et al. (2012) the regression was performed only on the average across trials. Because data from the *lag-9* condition were used to compute the regressor, they were not able to investigate results for the *lag-9* condition, nor investigate within-subject effects. In addition, this technique involves fixed spatial templates, so it is not suitable for revealing the time course of an experimental effect.

#### EMS filtering versus Fisher’s linear discriminant

EMS filtering is a matched spatial filtering technique: if one is testing for a difference between two conditions, then we compute the difference between their means and use that as a filter (or template). Fisher’s linear discriminant (FLD) is a well-known technique that operates in a similar way. In order to compute the weight vector for separating two classes, FLD takes the difference between the means of the two classes, and then multiplies this by the inverse noise-covariance matrix. The latter step can improve the separability of the two classes by taking account of non-uniformities in the noise distribution. However when the signal-to-noise ratio is low, trying to account for both signal and noise can yield a weight vector that is not representative of the difference between them.

A tradeoff between separability of the classes and recovery of the true underlying features (i.e. interpretability) is common among machine-learning techniques [29]. This tradeoff is primarily a function of the SNR, number of training samples, and level of correlation among the features. In the case of FLD, the dimensionality of the features is also a factor: For high-dimensional problems, it can become difficult to interpret the discriminative pattern because the inverse covariance estimate is especially sensitive to outliers [1]. As a general rule, an estimate of the “forward model” (sometimes referred to as a “scalp projection” in EEG) [1] will be more representative of the spatial distribution of activity over the sensor array. However, in the simple case where *w* = *f*(*X*,*y*) does not take the noise covariance into account, the weight vector and the forward model are identical.

For the analysis of stimulus-evoked responses, it is convenient for both the topography of the weight vector, and the resulting time course to be directly interpretable in terms of the particular experimental effect under investigation. Thus we have not incorporated the noise covariance into EMS filtering by default (although the option is available in our MatLab toolbox). Given that EMS filtering yields a significant improvement in signal quality over the most commonly-used techniques (single-sensor and ROI), foregoing an additional margin of separability at the single-trial level is a reasonable compromise in favor of readily interpretable weight vectors. Computing the noise covariance matrix also lengthens the computation time, especially in the context of a LOO cross validation.

#### EMS filtering versus pattern classification

Technically speaking, EMS filtering is a method of dimensionality reduction, and is not, by itself, a pattern classifier. However, it can be used for pattern classification by applying a simple decision rule (e.g. nearest-mean or GNB) to its one-dimensional output. In Figure 2C we compared the performance of this procedure (using a GNB decision rule) with that of a linear support-vector machine (SVM). The prediction accuracy of EMSf-GNB and the SVM were comparable, although the SVM consistently performed slightly better. Given this observation, one might ask whether EMS filtering carries any advantage over a pattern classification approach: i.e. one could examine the time course of prediction accuracy of the classifier, or examine the projection of the data onto the weight vectors that are learned on each round of cross-validation. There are three principal advantages of EMS filtering over pattern classification – speed, interpretability, and versatility – that should, for purposes of studying evoked responses, be weighed alongside classification accuracy.

The decoding approach requires that the performance of the decoder be estimated at each time point in the epoch, and this can be computationally expensive given that a cross validation must be performed for each estimate. Even with only a five-fold cross validation (far fewer rounds of cross validation than the leave-one-out procedure used by EMS filtering) the SVM still took, on average, more than 30 times longer to compute than EMS filtering (~ 1 – 2 minutes per subject for the SVM versus ~ 2 – 3 seconds per subject for EMS filtering; see Results / Performance).

Also, while the SVM yields slightly better separability of the two classes at the single-trial level, this may come at the expense of the interpretability of the topography of the weight vectors, as discussed above. In addition to the weight vectors, the time courses output from EMS filtering are also more readily interpretable. For a simple objective function such as the difference between the means of two conditions, the axis along which the surrogate time courses vary is simply (and exactly) *“the axis defined by the difference between the means of the two conditions in this data set”* – in the original measurement units. The same cannot be said of a time course expressed in units of “percent correct classification”, even in the context of a two-class problem.

**Figure 6:**
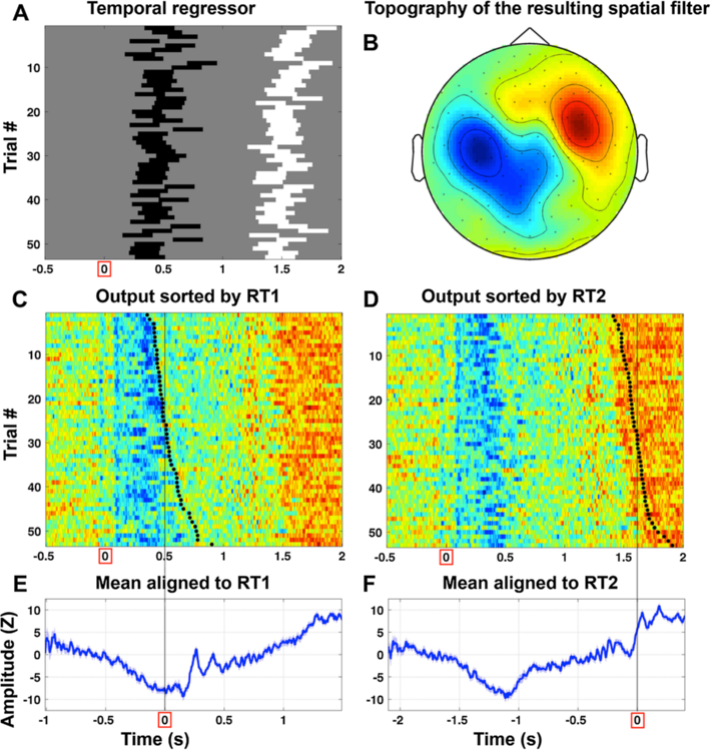
**EMS filtering with linear regression using a temporally defined predictor based on reaction-time data, applied to a single subject.** For this analysis, the objective function used by EMS filtering performed a linear regression on the data from each sensor (i.e. a matrix of trials x samples), and returned the beta weight. The predictor variable (shown in panel **A)** was constructed by coding each sample with a -1 if it was in the range 200 ms before to 50 ms after RT1, a +1 if it was in the range 200 ms before to 50 ms after RT2, and a zero otherwise. Since task 1 responses and task 2 responses were made with opposite hands (left and right, respectively, for this particular subject) then this regressor should reveal response-related activity that is different for right-handed and left-handed responses. The topography of the resulting spatial filter (magnetometers) is shown in panel **B**, and is clearly lateralized, consistent with the coding of the regressor. Panels **C** and **D** show the surrogate time courses sorted by RT1 and RT2, respectively, with the reaction time marked by black dots. The color map goes from blue (negative) to green (zero) to red (positive). Response-related activity is plainly visible in the form of a bluish vertical band at ~ 100 to 600 ms and a reddish vertical band at ~ 1400 to 2000 ms, and shows a clear relationship with the reaction time by which the data were sorted. Panels **E** and **F** show the mean over the surrogate time courses when the trials were aligned to RT1 and RT2, respectively. Data were arbitrarily aligned to the median reaction time in each case, which is marked by a thin vertical line. A confidence boundary equal to one standard error of the mean is shown in a lighter shade of blue.

**Figure 7:**
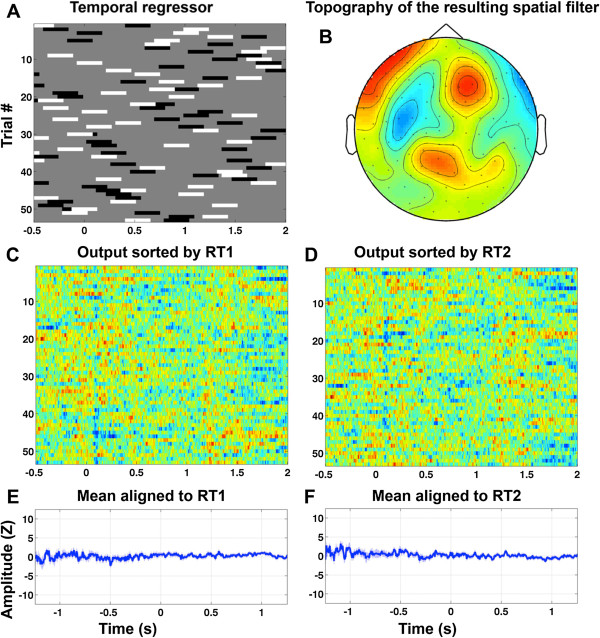
**Same as Figure**6**, but with the reaction times replaced by random values, resulting in a random model. A**, predictor variable (same as in Figure 6, but scrambled). **B**, topography of the resulting spatial filter. **C**, output sorted by RT 1; **D**, output sorted by RT2; **E**, mean aligned to RT1; **F**, mean aligned to RT2.

Finally, the pattern-classification approach (i.e. examining the time course of classification accuracy) is limited to questions concerning the difference between experimental conditions, which is only one possible objective function that can be applied using EMS filtering. Using EMS filtering one could, for example, project the data onto “the correlation between signal amplitude and reaction time” or “the difference in signal amplitude between t0-50 ms and t0-500 ms” (where t0 is the time of a motor response). The latter objective function would tend to capture activity that is changing prior to a movement, such as the readiness potential [10]. In the present treatment we have focused on the difference between means as an objective function, but we provide one example of the use of a different objective function in Figures 6 and 7. Note however that, pending a more general proof, optimality of the procedure for any function other than the difference between means cannot be guaranteed. We note again, however, that “not proven optimal” is not the same as “not useful”, especially when a provably optimal method with the same capabilities is either not known or does not exist.

#### *EMS filtering versus global field power (GFP)*[19]

EMS filtering applied to the difference between means gives as a solution the square-root of the total power of the ERP difference, and is thus very similar to the GFP of the ERP difference. However, GFP of the ERP difference is strictly an aggregate measure, and is not defined at the single-trial level, whereas EMS filtering produces one noise-suppressed time course per trial, and thus allows for both aggregate and single-trial analyses.

### Topography of the spatial filters

One might also want to ask “what sensors in the topography show a significant effect?”, but this is a different question from the one addressed by EMS filtering. In the context of EMS filtering, the significant effect, if there is one, is in the amplitude of the resulting time course at a particular time point – the “topography” at that time point is simply the vector onto which the original data were projected in order to reveal that significant effect. To make this point more clear, consider that it is possible to construct a topography such that all coefficients have the same absolute value (no sensor being weighted any more or less heavily than any other), and yet the vector of coefficients exposes a significant effect when the data are projected onto it. One could, however, statistically compare the spatial filter associated with a given experimental effect to that associated with a different effect (using correlation, for example), and one can also compare the spatial filter at a given time point with the spatial filter at a different time point, as illustrated in Figure 1C. To sum up this point, in the context of EMS filtering the approach is to make an assertion of the following form: “here we find a significant effect, and here we show the topography of the spatial filter that revealed the effect.”

It can, of course, be informative to examine the temporal evolution of the spatial filters. In Additional file 1 we illustrate some analyses for visualizing the stability of the spatial patterns over time and highlighting transitions between stable topographical patterns (c.f. D Lehmann, H Ozaki and I Pal [27]). One could also apply inverse source-modeling to the average spatial filter within a time-window of interest, but an appropriate method would have to be chosen that is valid when applied to a derived measure, such as the difference between two conditions.

### Contribution of EMS filtering to the dual-task literature: testing the sensory buffer hypothesis

The router model of the PRP [7] proposes the existence of a sensory buffer whose properties are related to the recurrent connections within a hierarchical sensory cortex. In a single-task situation, the target stimulus is processed in the sensory cortex and rapidly accesses the serial central stage. In a dual-task situation, T2 triggers the activation of the sensory buffer, but it cannot access the central stage before the completion of task 1. Thus, according to the model, T2 sensory information slowly decays until a decision is reached for task 1. Testing this hypothesis directly can be done by examining T2 sensory integration within subjects at the single trial level. Once T2-related activity was isolated for each trial, it was possible to decompose this activity into different events and to look at the properties of each one of these. We found that the duration of the early event, but not its latency, was influenced by RT1. Conversely, the latency but not the duration of the late event was influenced by RT1. These results suggest that the information related to T2 is held in a sensory buffer until the response to T1 is executed. Once the motor response to T1 is executed, T2 information can access a second stage which is strictly serial (i.e. delayed during the PRP), and absent in unseen trials (Marti et al., 2012).

### Objective functions other than the difference between means

Direct comparison between two experimental conditions is a very simple, common, and powerful analysis, and this is why we have focused on the difference between means as an objective function for the purpose of demonstrating the method. However, as an algorithm EMS filtering is independent of the particular objective function that is used. The minimum requirement is that the function return a vector of coefficients, one for each sensor. As one extreme (and useless) example, the objective function could return a vector of random numbers – highly unlikely to reveal any interesting effects in the data, but technically a valid objective function. Correlation with a behavioral variable such as reaction time is an example of a potentially informative objective function. Although in principle any objective function can be used, note that the interpretability of the results will depend, at least in part, on the choice of objective function. Note also that the magnitude and direction of the skew introduced by the LOO procedure [9] might not be the same for all objective functions, but, again, this is only a concern when doing statistical testing on data from a single subject, in which case a resampling test should be performed anyway.

In order to illustrate the use of EMS filtering with an objective function other than the difference between means, we used the reaction-time data from [5] to construct a temporal regressor, and then applied an objective function that returned the fit, at each sensor, of this regressor to the MEG data. The temporal regressor highlighted the time interval from -200 ms to +50 ms with respect to each response, with the opposite sign (-1/+1) for responses made with the left and right hands, respectively (responses to task 1 were all made with the same hand, and responses to task 2 were always made with the opposite hand, with the assignment of hands counterbalanced across subjects). We anticipated that this would reveal motor-related activity and highlight the difference between right- and left- hand responses, and the results are striking (Figures 6 and 7).

## Conclusions

We have presented a method for reducing multi-variate time series data to a single time course by projecting the data onto a single vector that is chosen so as to reveal a given experimental effect. A leave-k-out procedure ensures that each filter is independent of the trial/s that is/are projected onto to it. Although we have presented the method primarily using the example of a difference between two experimental conditions, we reiterate that other objective functions – such as correlation with a dependent or independent variable, or a temporal difference within a single experimental condition – can also be used, and this capability has been implemented in the freely-available computer code. However, the properties of any function other than the difference between two means would have to be worked out independently. We used MEG data to illustrate the method, but the method can be applied to any kind of multivariate time-series data, such as slow event-related functional magnetic resonance imaging (fMRI), or data from domains outside of neuroimaging. We have demonstrated the effectiveness of the method in dramatically improving the quality of single trial data vis-à-vis a given experimental effect, and specifically in revealing the time course of the psychological refractory period. An implementation of the method is freely available as a MatLab toolbox at http://bitbucket.org/emsf/emsf_matlab.

## Endnotes

^a^A clarification on the use of the term "spatial filter": A spatial filter can be two-dimensional, taking into account the relative spatial locations of each of the individual variables (pixels or sensors). In this case a two-dimensional convolution is used for filtering, as in the example of detecting faces in photographs. However, even when we treat the data as a one-dimensional vector (as is the case here), the term "spatial filter" is still commonly used because the data (across sensors, at a single time sample) are in the spatial domain rather than the time domain. When we refer to a "spatial filter" we are always referring to a one-dimensional vector in sensor space (and hence a one-dimensional filtering operation).

^b^One can show analytically, that the expected mean of this LOO procedure is zero. However, for low dimensional data (D<10) the distribution is far from the Normal distribution and thus one can not use the conventional t-test for the mean (as the variance will be over-estimated and thus one looses statistical power). Non-parametric tests for zero-median are also not adequate as the distribution is skewed and thus it has a non-zero median. To establish statistical significance one has to resort therefore to random shuffle statistics. The problem may be less severe for high-dimensional data in which case the distribution is approximately Normal and a simple t-test may suffice. (LC Parra, personal communication). [Note that the above is not a concern when performing statistics across subjects, as long as the means are normally distributed].

## Appendix A

EMS filtering – formal description

Abbreviations and notation

In all formulae, ***X***._*j*_ refers to the j^th^ column of matrix ***X***, and *X*_*i.*_ refers to the i^th^ row. Where **X** is a three-dimensional data matrix, ***X***_..k_ is equivalent to “every row and every column where the third index is *k*”, or more formally ∀*i*, ∀*j*, ∃*k****X***_*ijk*_.

The EMS filtering algorithm

Let ***X*** be a data matrix of size *m* x *n* x *p*, where *m* is the number of sensors, *n* is the number of samples in each epoch, and *p* is the number of trials. These are indexed by *i*, *j*, and *k*, respectively. There is also a vector *y*, of condition labels of size *p* x 1 (i.e. one for each trial). Let *f* be a function that takes as parameters the data at a single time point in ***X*** (i.e. ***X***_***.j.***_, a 2-D matrix of size *m* x *p*), and a set of condition labels, *y*, of size *p* x 1, and returns a set of coefficients of size *m* x 1, i.e. one per sensor. We refer to *f* as the “objective function”, since we are interested in maximizing this function over the output of the algorithm.

If the objective is to maximize the difference between two experimental conditions, then *f* simply computes the difference between the average (across trials) for condition *y* = 1 and the average (across trials) for condition *y* = 2. This difference is computed individually for each sensor. Each element in the resulting vector, *d*, can be thought of as a coefficient whose sign and magnitude correspond to the sign and magnitude of the difference between conditions *y* = 1 and *y* = 2 at that sensor, at that time point. In the event that different sensors have different units (as with gradiometers and magnetometers in MEG), then either the data must be converted to Z scores (separately for each subset of sensors) *prior to* EMS filtering, or data from the different sensor types must be filtered separately. Since we will use *d* as a spatial filter, we set it to unit length by dividing by its norm (the square root of the sum of the squared values), in order to preserve the units of the data that will be projected onto it.

Given only the data matrix, *X*, and the condition labels, *y*, the most appropriate spatial filter for revealing a given experimental effect at time *t* is the one derived based on the data at time *t*. Hence we define a new function, *F*, that simply computes *f* independently at each time point in *X*, resulting in *a distinct spatial filter for each time point in the trial epoch* (Figure 1C). Thus the function *F*, instead of returning a single vector *d*, returns a matrix *D*, where each column is a spatial filter (*d*^*t*^) derived based on the data at the corresponding time point (*t*) in *X*.

We could simply compute *D* based on *X* and then project the data at each time sample of each trial in *X* onto its corresponding column in *D*, resulting in one time course per trial.

$$D=F\left(X,y\right)$$

$$S_{\mathrm{jk}}=\underset{i}{\sum }D_{\mathrm{ijk}}X_{\mathrm{ijk}}$$

Each time point (*t*) in each resulting time course in *S* would reflect the projection of the data onto the mean difference between condition *y* = 1 and *y* = 2 at time *t* in our data. However, this would introduce circularity in the data analysis, a common error that can lead to exaggerated or spurious experimental effects [8,9]. This is because the data that are projected onto each template in the set are the same data used to derive the template. In order to avoid this, we iteratively replace each trial with its projection onto the spatial filter derived based on all of the *other* trials – a simple *leave-one-out* procedure. If the number of trials per condition is balanced, then a *leave-one-out-per-condition* (LOOPC) procedure can be used, and if the algorithm is applied across subjects, rather than within subject, then a *leave-one-out-per-subject* (LOOPS) procedure should be used. [Note that although the LOO procedure may give the best estimate of the template for filtering the left-out trial, it may not be optimal vis-à-vis aggregate measures applied to the resulting time courses. The average (of averages) over a five- or ten-fold cross validation may give a better estimate (our MatLab implementation allows for k-fold cross validation).]

For the simple case where *f* takes the difference between the means of two experimental conditions, the formal solution is as follows:

Let

(1) Mijk1=∑k'∈cond1k'≠kn1Xijk'n1

be the mean for condition ***y*** = 1, with the *k*^*th*^ trial left out, where *n1* is the number of trials belonging to condition ***y*** = 1.

Let

(2) Mijk2=∑k'∈cond2k'≠kn2Xijk'n2

be the mean for condition ***y*** = 2, with the *k*^*th*^ trial left out, where *n2* is the number of trials belonging to condition ***y*** = 2.

Then

(3) $$D_{\mathrm{ijk}}=M_{\mathrm{ijk}}^{1}-M_{\mathrm{ijk}}^{2}$$

is the matrix of spatial filters, one for each sample (*j*) of each trial (*k*),

$$\hat{D}._{\mathrm{jk}}=D._{\mathrm{jk}}/∥D._{\mathrm{jk}}∥$$

is the matrix of spatial filters after normalizing each filter to unit length, and

(4) $$S_{\mathrm{jk}}=\sum_{i}{\hat{D}}_{\mathrm{ijk}}X_{\mathrm{ijk}}$$

is the resulting matrix of surrogate time courses.

For the general case where ***f*** is some function other than the above (see Discussion), *for each* k∈[1,2,…,*p*] we let *X*^*(k)*^ = *X* with the *k*^*th*^ trial left out and let *y*^*(k)*^ = *y* with the *k*^*th*^ trial-label left out, and define S_*k*_, the *k*^*th*^ surrogate time course, as follows:

(5) $$S_{k\cdot }=\left[\sum_{i}X_{\mathrm{ijk}}{\hat{D}}_{\mathrm{ijk}}\right]$$

Where

$${\hat{D}}_{\cdot \mathrm{jk}}=D_{\cdot \mathrm{jk}}/∥D_{\cdot \mathrm{jk}}∥$$

and

(6) $$D_{\cdot \cdot k}=f\left(X^{\left(k\right)},y^{\left(k\right)}\right)$$

Recall the formal definition of a matched filter as a “known template” that we correlate with an unknown (and noisy) signal. In the context of EMS filtering we do not know what the template is (i.e. the real underlying difference between two experimental conditions), and so we estimate it from the data itself. This means that performance of the filter will depend in part on the accuracy of the estimate. If the estimate is optimal, then this is the best that can be done *given only the data at hand* (without using a more nuanced model of the noise). For the difference between two means the estimate is known to be optimal a-priori, and so iid-optimality follows by virtue of this being a matched filter. For objective functions other than the difference between two means, the procedure may or may not be iid-optimal, contingent on the accuracy of the estimate of the template. Our MatLab implementation uses function handles (i.e. pointers) in order to support user-defined objective functions. This allows one to perform analyses that would be difficult or impossible using other methods (see Figures 6 and 7).

Filtering with a stationary-template

Although we present EMS filtering as a method for revealing the time course of an experimental effect, it is often useful to examine the time course of a fixed spatial filter e.g. the mean around the peak of an evoked potential of interest. While conceptually different from the procedure described above, its implementation is very similar. Consider an experimental effect that is expected to appear at a specific latency (e.g. ~200 ms) with respect to a certain event – e.g. the onset of a stimulus or the issuance of a motor response – to which the data epochs are aligned (*t0*). We define a temporal window of interest, *w*, which is a list of sample indices in our data epochs corresponding to, for example, +180 to +220 ms with respect to *t0*. *f* is a function that takes as parameters the data matrix, ***X***, the set of trial labels, *y*, and the list of sample indices, *w*, and returns a set of coefficients of size *m* x 1 (i.e. one per sensor). In the simplest case, *f* simply takes the average over the temporal window specified by *w*, and then returns the difference between the average (across trials) for condition *y* = 1 and the average (across trials) for condition *y* = 2.

So *for each k ∈ {1,2,…,p*}, we let ***X***^*(k)*^ = ***X*** with the *k*^*th*^ trial left out and *y*^*(k)*^ = *y* with the *k*^*th*^ trial-label left out, and define S_*k*_ , the *k*^*th*^ surrogate time course, as follows:

(7) $$S_{k.}={\left[{\left[X.._{k}\right]}^{T}•{\hat{d}}^{k}\right]}^{T}$$

Where ${\hat{d}}^{k}=d^{k}/∥d^{k}∥$ and

(8) $$d^{k}=f\left(X^{\left(k\right)}{}_{'}y^{\left(k\right)}{}_{'}w\right)$$

is the spatial filter computed on the *k*^th^ iteration. In the example given above, *f* simply computes the difference between the mean of trials belonging to condition *y* = 1 and the mean of trials belonging to condition *y* = 2, in the time window specified by *w*. Although the algorithm itself is independent of the specific function that is used, we reiterate that the performance of the procedure is contingent on the accuracy of the estimate that the function computes.

Since the average signal at time(s) t ∈ *w* was used to derive the spatial filter *d*, then projection onto *d* is an appropriate choice for maximizing the separation between the mean signal amplitude for condition *y* = 1 versus condition *y* = 2, *at time(s) t ∈ w*. If there is in fact a detectable difference in the data at time(s) *t ∈ w*, then we expect it to be apparent in the average over the surrogate measures in that same time window. If a difference is also apparent at some *other* time in the trial, this would imply that a very similar topography also appears at this time in the course of the trial. Thus, in addition to being useful for estimating the latency of a signal event at the single-trial level (Figure 5), this approach can also be used to reveal the re-appearance of a particular topographical template at different times in the trial epoch [5]. These are two inferences that one can reasonably draw when using a fixed (stationary) template for the entire trial epoch. To follow the time-course of an experimental effect, a different template should be computed for each time point in the trial epoch.

## Appendix B

Derivation of the weight vector for the difference between two means

In the Methods section we introduced ***X***, the data matrix, as having dimensions *m* x *n* x *p*, where *m* is the number of sensors, *n* is the number of time samples in the epoch, and *p* is the number of epochs (or trials). Since the fundamental operation of EMS filtering is independent for each time sample in the epoch, then for the mathematical derivation we consider a matrix *X* with only one time sample, i.e. with dimensions *m* x *p* (# of sensors by # of trials).

*y* is a vector of trial labels of size *p* x 1, where *y* = 1/*N*_*A*_ if the corresponding trial belongs to condition *A*, and *y* = -1/*N*_*B*_ if the corresponding trial belongs to condition *B*, and *N*_*A*_ and *N*_*B*_ are the number of trials belonging to conditions *A* and *B*, respectively.

(9) $$\text{Find the vector}w=\underset{ws.t.∥w∥=1}{\mathrm{argmax}}\sum_{k}y_{k}\langle X_{k}{}_{,}w\rangle$$

Where ⟨χ ,y⟩ denotes the scalar product, or dot product, often written as *χ*. *y*.

The straightforward solution to this problem is:

(10) $$\hat{w}=\frac{\sum_{k}y_{k}X_{k}}{∥\sum_{k},y_{k}X_{k}∥}$$

Note that the numerator in (10) above is equivalent to the vector labeled *d* in section 2.1.1 (i.e. the difference between the mean over condition *A* and the mean over condition *B*), and when repeated for each time sample in the epoch is equivalent to the matrix labeled *D* in Appendix A, equation (3). Note also the norm of *w* plays no role in the problem – it is simply a global scaling factor, and must simply be fixed at some value. You can thus fix ||*w*|| = 1 without loss of generality. Having ||*w*|| = 1 is useful in order to preserve the measurement units in the output.

Cross validation

The accuracy of the model is simply the dot product of the discriminative vector *w* with the weighted observations,

$$\left(y,X\right)\to \sum_{k}y_{k}\langle X_{k}{}_{,}w\rangle$$

To discount over-fitting effects in the accuracy estimate, we replace it by a cross-validated estimate,

$$f=\sum_{k}y_{k}\langle X_{k}{}_{,}w^{\left(k\right)}\rangle$$

where *w*^*(k)*^ = *w* derived as above, but based on *X*^*(k)*^ and *y*^*(k)*^, where the superscript *(k)* means “with the *k*^*th*^ trial left out”.

## Competing interests

The authors declare that they have no competing interests.

## Authors’ contributions

AS devised the method and wrote the MatLab computing toolbox. SM contributed computer code to the toolbox. AS and SM tested and refined the method. AS and SM performed the data analyses. AS, SM, and SD wrote the manuscript, and all authors read and approved the final manuscript.

## Supplementary Material

Additional file 1 Projection onto the difference between two means with LOO cross--- validation on guassian random data. Click here for file

Additional file 2 Visualizing the temporal evolution of the spatial filters. Click here for file

## References

[B1] Parra LC Spence CD Gerson AD Sajda P Recipes for the linear analysis of EEG Neuroimage 2005 14 2 326 341 10.1016/j.neuroimage.2005.05.032 16084117

[B2] Parra L Alvino C Tang A Pearlmutter B Yeung N Osman A Sajdal P Linear spatial integration for single-trial detection in encephalography Neuroimage 2002 14 1 223 230 10.1006/nimg.2002.1212 12482079

[B3] Turin GL An Introduction to Matched Filters IRE Trans Info Theory 1960 14 3 311 329 10.1109/TIT.1960.1057571

[B4] Brunelli R Poggio T Template Matching: Matched Spatial Filters and Beyond Pattern Recognit 1995 14 5 751 768

[B5] Marti S Sigman M Dehaene S A shared cortical bottleneck underlying Attentional Blink and Psychological Refractory Period Neuroimage 2012 14 3 2883 2898 10.1016/j.neuroimage.2011.09.063 21988891

[B6] Pashler H Dual-Task Interference in Simple Tasks: Data and Theory Psychol Bull 1994 14 2 220 244 7972591 10.1037/0033-2909.116.2.220

[B7] Zylberberg A Fernandez Slezak D Roelfsema PR Dehaene S Sigman M The brain's router: a cortical network model of serial processing in the primate brain PLoS Comp Biol 2010 14 4 e1000765 10.1371/journal.pcbi.1000765 PMC2861701 20442869

[B8] Kriegeskorte N Simmons WK Bellgowan PSF Baker C Circular analysis in systems neuroscience: the dangers of double dipping Nat Neuro 2009 14 5 535 540 10.1038/nn.2303 PMC2841687 19396166

[B9] Vul E Harris C Winkielman P Pashler H Puzzlingly High Correlations in fMRI Studies of Emotion, Personality, and Social Cognition Perspect Psychol Sci 2009 14 3 274 290 10.1111/j.1745-6924.2009.01125.x 26158964

[B10] Shibasaki H Hallett M What is the Bereitschaftspotential Clin Neurophysiol 2006 14 11 2341 2356 10.1016/j.clinph.2006.04.025 16876476

[B11] Pedregosa F Varoquaux G Gramfort A Michel V Thirion B Grisel O Blondel M Prettenhofer P Weiss R Dubourg V Scikit-learn: Machine Learning in Python J Mach Learn Res 2011 14 2825 2830

[B12] Oostenveld R Fries P Maris E Schoffelen JM FieldTrip: Open Source Software for Advanced Analysis of MEG, EEG, and Invasive Electrophysiological Data Comput Intell Neurosci 2011 2011 Article ID 156869, 9 pages, doi:10.1155/2011/156869 10.1155/2011/156869 PMC3021840 21253357

[B13] Bell A Sejnowski TJ An information-maximization approach to blind separation and blind deconvolution Neural Comput 1995 14 6 1129 1159 10.1162/neco.1995.7.6.1129 7584893

[B14] Hyvärinen A Oja E Independent component analysis: algorithms and applications Neural Netw 2000 14 4–5 411 430 10946390 10.1016/s0893-6080(00)00026-5

[B15] Jung T-P Makeig S Humphries C Lee T-W McKeown M Iragui V Sejnowski TJ Removing electroencephalographic artifacts by blind source separation Phsychophysiol 2000 14 163 178 10.1111/1469-8986.3720163 10731767

[B16] Fairhall AL Burlingame CA Narasimhan R Harris RA Puchalla JL Berry MJ 2nd Selectivity for multiple stimulus features in retinal ganglion cells J Neurophysiol 2006 14 5 2724 2738 10.1152/jn.00995.2005 16914609

[B17] Brenner N Bialek W De Ruyter Van Steveninck R Adaptive rescaling maximizes information transmission Neuron 2000 14 3 695 702 10.1016/S0896-6273(00)81205-2 10896164

[B18] McIntosh AR Bookstein FL Haxby JV Grady CL Spatial pattern analysis of functional brain images using partial least squares Neuroimage 1996 14 3 Pt 1 143 157 9345485 10.1006/nimg.1996.0016

[B19] Skrandies W Global Field Power and Topographic Similarity Brain Topogr 1990 14 1 137 141 10.1007/BF01128870 2094301

[B20] Sigman M Dehaene S Brain mechanisms of serial and parallel processing during dual-task performance J Neurosci 2008 14 7585 7598 10.1523/JNEUROSCI.0948-08.2008 18650336 PMC6670853

[B21] Hesselmann G Sadaghiani S Friston KJ Kleinschmidt A Predictive coding or evidence accumulation? False inference and neuronal fluctuations PLoS ONE 14 3 e9926 10.1371/journal.pone.0009926 PMC2848028 20369004

[B22] de Cheveigné A Simon JZ Denoising based on spatial filtering J Neurosci Methods 2008 14 2 331 339 10.1016/j.jneumeth.2008.03.015 18471892 PMC2483698

[B23] Särelä J Valpola H Denoising Source Separation J Mach Learn Res 2005 14 233 272

[B24] Koles Z The quantitative extraction and topographic mapping of the abnormal components in the clinical EEG Electroenceph Clin Neurophysiol 1997 14 6 440 447 10.1016/0013-4694(91)90163-x 1721571

[B25] Owen F Kenneth PC Joseph M The analytic common spatial patterns method for EEG-based BCI data J Neural Eng 2012 14 4 045009 10.1088/1741-2560/9/4/045009 22832090

[B26] de Cheveigne A Simon JZ Sensor noise suppression J Neurosci Methods 2008 14 1 195 202 10.1016/j.jneumeth.2007.09.012 17963844 PMC2253211

[B27] Lehmann D Ozaki H Pal I EEG alpha map series: brain micro-states by space-oriented adaptive segmentation Electroenceph Clin Neurophysiol 1987 14 271 288 10.1016/0013-4694(87)90025-3 2441961

[B28] Hesselmann G Flandin G Dehaene S Probing the cortical network underlying the psychological refractory period: A combined EEG-fMRI study Neuroimage 2011 14 3 1608 1621 10.1016/j.neuroimage.2011.03.017 21397701

[B29] Hastie T Tibshirani RJ Friedman R The Elements of Statistical Learning 2008 New York, NY: Springer-Verlag DOI-10:0387848576

