# Supplementary material for: Reducing multi-sensor data to a single time course that reveals experimental effects
Source: BMC Neurosci. 2013 Oct 14;14:122. doi: 10.1186/1471-2202-14-122 (PMC4015840; doi:10.1186/1471-2202-14-122)
Supplement: Additional file 1 — Projection onto the difference between two means with LOO cross--- validation on guassian random data. [file 1471-2202-14-122-S1.pdf]

Projection onto the difference between two means with LOO cross-validation on guassian random data

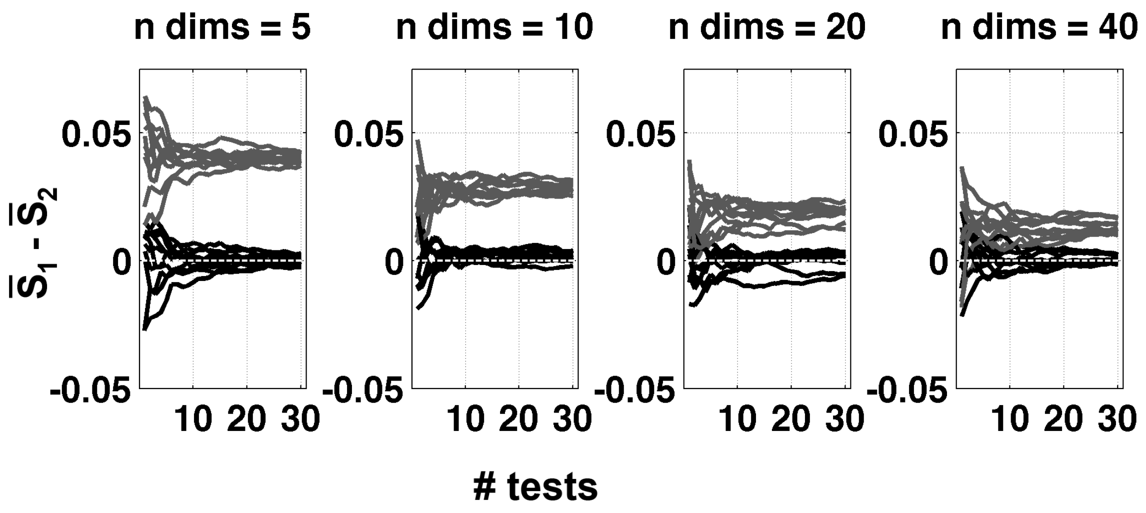

Figure A2: Projection onto the difference between condition means with LOO cross-validation on gaussian random data: zero mean, but not zero median.

The mean of each simulated experiment is shown in black, and the median is shown in gray. Gaussian random data were generated with the number of “sensors” (i.e. dimensions) shown above each plot. Each simulated experiment consisted of 64 trials (32 per condition). On each iteration of the LOO procedure, two trials were left out – one from each condition. These were projected onto the difference between the means of the two conditions at each “sensor”, estimated from the remaining trials (with the projection vector scaled to unit length). For each experiment, we then computed the difference between the means of the two conditions over the projected time courses. Each point in the plots above represents the average over 1000 experiments. This entire procedure was repeated for 10 series of 30 repetitions. Each of the traces in the figures above represents the cumulative mean over one of the 10 series. Note that the means (black) converge to zero, but the medians (gray) converge towards some value greater than zero, depending on the number of dimensions. The means (over the differences between means) were not detectably different from zero after correcting for four comparisons ($p = 0.48, 0.11, 0.47$, and 0.43, uncorrected, for 5, 10, 20, and 40 dimensions, respectively, using a two-tailed t-test, and taking the average p value over 1000 bootstrap sample). The medians were significantly different from zero in all four cases, using either a two-tailed t-test or two-tailed signed-rank test.
