# Supplementary material for: Reducing multi-sensor data to a single time course that reveals experimental effects
Source: BMC Neurosci. 2013 Oct 14;14:122. doi: 10.1186/1471-2202-14-122 (PMC4015840; doi:10.1186/1471-2202-14-122)
Supplement: Additional file 2 — Visualizing the temporal evolution of the spatial filters. [file 1471-2202-14-122-S2.pdf]

Visualizing the temporal evolution of the spatial filters

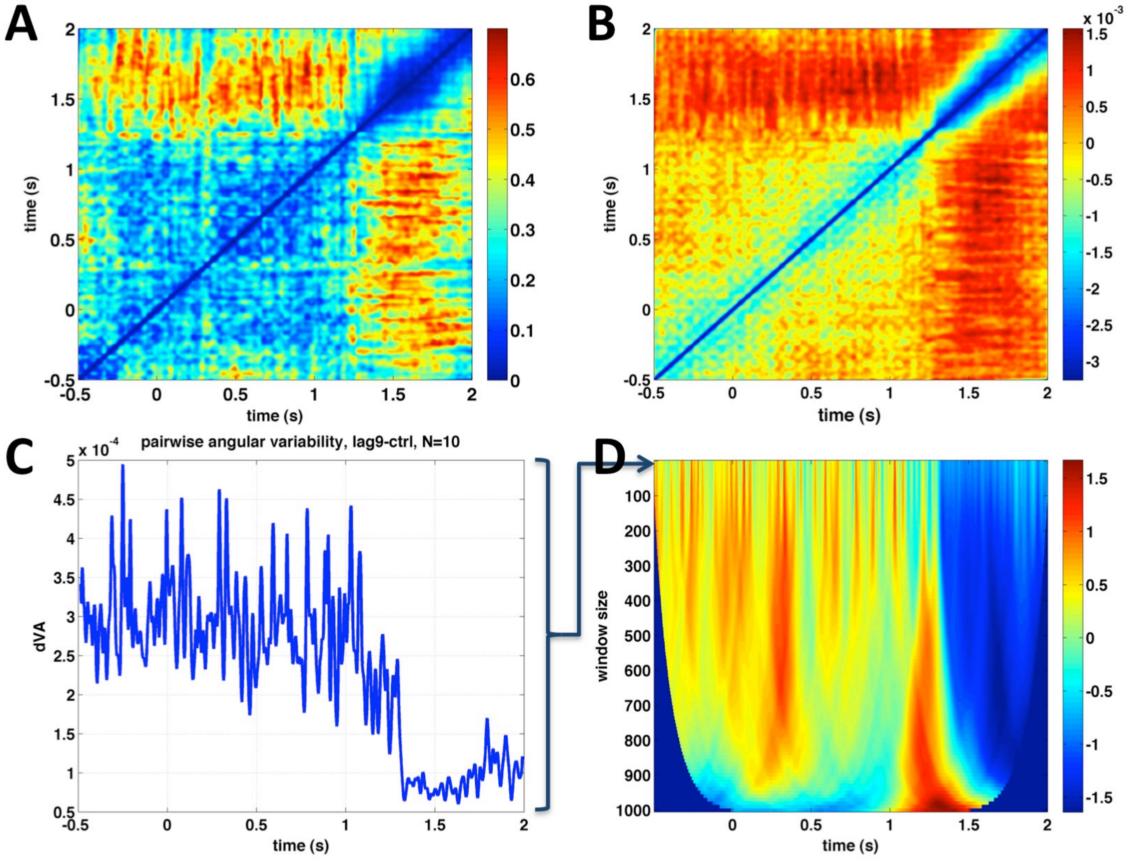

Figure A1: Analyses applied to the temporal evolution of the spatial filters from the standard version of EMS filtering (average across 10 subjects).

(**A**) Pairwise angular dispersion between the spatial filter at each time point and the spatial filter at every other time point (symmetrical about the diagonal). (**B**) The sign-reversed covariance matrix of the matrix of spatial filters (also symmetrical about the diagonal). We reverse the sign for visual comparison with panel **A**. (**C**) Pairwise angular dispersion between temporally adjacent pairs of spatial filters (i.e. within a sliding window of size 2). (**D**) Mean angular dispersion within sliding windows of size *n*, with *n* varied from 2 (i.e. the time course shown in **C**) to 1000. Each of these was computed individually for each subject, and then averaged across subjects to produce the results shown here.

For the set of vectors $v_1, v_2, \ldots, v_n$ the angular dispersion is given by $1 - \|\sum \hat{v}_1 + \hat{v}_2 + \cdots + \hat{v}_n\|/n$, where $\hat{v} = \frac{v}{\|v\|}$. Intuitively, the angular dispersion gives the degree to which the set of vectors are all pointing in the same direction, i.e. the degree to which the set of vectors resemble one another. To the extent that the vectors are all pointing in the same direction, then angular dispersion will tend toward zero, and will tend towards 1 when the vectors are pointing in random directions. In panel **A** above, the window of time between ~ 1.2 to 2.0 s shows the signature of a stable topography that is different from the topography at any point in time prior to that time window: The red band across the top, extending out to ~

1.2 s, indicates that the spatial pattern within this window of time is relatively unlike any before it. The blue expansion around the diagonal in the upper right indicates that the spatial patterns within this time window are relatively similar to one another, and remain so across this time interval. The covariance matrix in panel **B** gives similar information, although pairwise angular dispersion appears to be more sensitive to fine structure in the time course. In addition to looking at pairwise angular dispersion between vectors at different, non-adjacent points in time, one can compute angular dispersion within a contiguous temporal window of size *n*, where *n* can vary from 2 to ½ the length of the time course. Panel **C** shows the angular dispersion within a sliding window of size 2. This becomes the top row in panel **D**, which shows the angular dispersion within a contiguous sliding window varying in width from 2 through 1000 (vertical axis). Colors tending toward red indicate relative variability in the angle of the vectors within the sliding window, i.e. fluctuations in the topography on the corresponding time scale. Colors tending toward blue indicate a relatively stable topography over the time window of a given width. Note the long period of relative stability from ~1.3 to 2.0 sec.
